# Telecardiologia e Seu Potencial em Áreas Remotas

**DOI:** 10.36660/abc.20230281

**Published:** 2023-06-16

**Authors:** Simone Farah

**Affiliations:** 1 Faculdade de Medicina de Petrópolis UNIFASE Rio de Janeiro RJ Brasil Faculdade de Medicina de Petrópolis – UNIFASE, Rio de Janeiro, RJ – Brasil

**Keywords:** Telemedicina, Telecardiologia, Teleconsulta, Doenças Cardiovasculares, Áreas Remotas, Prevenção de Doenças

Muitas foram as mudanças ocorridas nos últimos anos no campo da Telemedicina, no Brasil e no mundo, para o enfrentamento da pandemia da COVID-19. Avançamos no entendimento da relevância e do impacto da telemedicina e sua aplicação nos cuidados em saúde, principalmente na prevenção e promoção da saúde. E para acompanhar todas essas transformações foi necessário também avançarmos do ponto de vista legal. No período da pandemia a Lei No. 13.989 de 15 de abril de 2020^1^ permitiu o atendimento direto ao paciente através da teleconsulta, algo que até então não era permitido no Brasil. Atualmente temos em vigência a Resolução de Telemedicina do Conselho Federal de Medicina No. 2.314 de 05 de maio de 2022^2^ e a Lei No. 14.510, conhecida como a Lei da Telessaúde, de 27 de dezembro de 2022^3^que permite a teleconsulta em todo o território nacional. Isso permitiu que áreas antes desprovidas de atendimento especializado pudessem ter acesso ao especialista em termo real (síncrono) para atendimento em diversas especialidades.

A teleconsulta médica, definida pela consulta não presencial, mediada por Tecnologias Digitais de Informação e Comunicação (TDIC), com médico e paciente localizados em diferentes espaços,^2^ é uma das modalidades de atendimento por telemedicina. Na cardiologia, essa modalidade se faz ainda mais importante uma vez que as doenças cardiovasculares (DCV) são a maior causa de morbimortalidade no Brasil e no mundo, com crescente importância à medida que ocorre o envelhecimento populacional, impactando em custos para os sistema de saúde.^4^ E em um país da dimensão territorial do Brasil, a presença do especialista em todos os locais se torna muito difícil, além de demandar muito tempo e alto custo para viabiliza-lo em todos os pontos de atendimento primário ou secundário.^5^

Nesse contexto, a prevenção e o diagnóstico precoce dos fatores de risco têm um papel crucial para reduzir a incidências das DVC.^6^ E a telecardiologia na modalidade teleconsulta em áreas desprovidas de acesso ao especialista tem o potencial de otimizar a identificação dos pacientes com maior risco para as DCV.

Accorsi et al.,^7^ no artigo publicado nos Arquivos Brasileiros de Cardiologia, analisaram os dados de teleconsultas cardiológicas realizadas em pacientes moradores de cidades remotas na região Norte do Brasil. Segundo o último Censo do Instituto Brasileiro de Geografia e Estatística, a Região Norte do Brasil tem mais de 12.500.000 habitantes, sendo que pelo menos 20% vivem em áreas remotas, distantes dos centros urbanos ou distantes de locais habitados, e de difícil acesso.^4^ A Região Norte tem a menor densidade de serviços médicos do país, com uma média de um médico por mil habitantes, mas chegando a 0,2 por mil habitantes em áreas remotas.^8^

O estudo^7^ envolveu o centro de telemedicina do Hospital Israelita Albert Einstein tendo sido referência para 104 centros de atendimento presencial na Região Norte do Brasil relacionados ao programa de assistência médica especializada do Programa de Apoio ao Desenvolvimento Institucional do Sistema Único de Saúde (PROADI) do Ministério da Saúde. Os pacientes foram previamente avaliados por médicos generalistas da comunidade que solicitaram consulta especializada em cardiologia. Todas as avaliações remotas deste programa incluíram o paciente ao lado do clínico geral da unidade de saúde com o cardiologista do centro de telemedicina em tempo real (síncrono). Dentre os parâmetros analisados pelos autores ressaltam-se, o motivo do encaminhamento, histórico clínico e exame físico e avaliação pós telemedicina quanto a exames, medicamentos, diagnóstico e prescrições. No período de 17/02/2020 a 04/10/2021, 653 pacientes foram agendados, com taxa de comparecimento às consultas de 85,7%.

Pontos a serem destacados na teleconsulta cardiológica:

- Principais sintomas que motivaram o encaminhamento: dor torácica, dispneia, palpitação e sincope.
- Em apenas 26,1% havia a suspeita de cardiopatia isquêmica
- Principais fatores de risco identificados: hipertensão arterial, dislipidemia, tabagismo, sedentarismo e diabetes mellitus.
- A maioria dos pacientes teve exame físico e eletrocardiograma normal.
- Todas as prescrições de medicamentos foram alteradas em alguma medida.
- Pouca necessidade de solicitação de exames complementares, com muito poucos pacientes indicados para intervenção.
- Muitos pacientes precisaram de acompanhantes para entender as explicações básicas do tratamento.

Esse foi o primeiro estudo a analisar as características e a gestão de consultas cardiológicas por telemedicina sob demanda para populações de baixa renda em áreas remotas do Brasil, demostrando o potencial da teleconsulta cardiológica para otimizar o encaminhamento ao especialista em áreas remotas e de difícil acesso na Região Norte e a oportunidade de otimizar o tratamento médico de diversas cardiopatias.
