# Supplementary material for: Telecardiologia e Seu Potencial em Áreas Remotas
Source: Arq Bras Cardiol. 2023 Jun 16;120(5):e20230281. [Article in Portuguese] doi: 10.36660/abc.20230281 (PMC12080600; doi:10.36660/abc.20230281)
Supplement: Translation

# Translation: Telecardiology and its Potential in Remote Areas

**Authors:** Simone Farah

**Affiliations:** 1 Brazil Faculdade de Medicina de Petrópolis – UNIFASE, Rio de Janeiro, RJ – Brazil

**Keywords:** Telemedicine, Telecardiology, Teleconsultation, Cardiovascular Diseases, Remote Areas, Disease Prevention

Many have been changes that have taken place in recent years in the field of Telemedicine, in Brazil and in the world, to face the COVID-19 pandemic. We made progress in understanding the relevance and impact of Telemedicine and its application in health care, especially in prevention and health promotion. Furthermore, to keep up with all these transformations, it was also necessary to move forward from a legal point of view. During the pandemic, Law No. 13,989 of April 15, 2020^1^ allowed direct patient care through teleconsultation, which was not allowed in Brazil until then. Currently, Telemedicine Resolution No. 2,314 of May 5, 2022,^2^ and Law No. 14,510, known as the Telehealth Law, of December 27, 2022,^3^ are in effect, which allows teleconsultation throughout the national territory. This allowed areas previously devoid of specialized care access to specialists in real terms (synchronous) to provide care in various specialties.

Medical teleconsultation, defined as non-face-to-face consultation, mediated by Digital Information and Communication Technologies (TDIC), with doctor and patient located in different spaces,^2^ is one of the modalities of telemedicine care. In cardiology, this modality becomes even more important since cardiovascular diseases (CVD) are the major cause of morbidity and mortality in Brazil and the world, with increasing importance as population aging occurs, impacting costs for the health system.^4^ And in a country the size of Brazil, the presence of specialists in all locations becomes very difficult, demanding a lot of time and high cost to make it available at all points of primary or secondary care.^5^

In this context, the prevention and early diagnosis of risk factors play a crucial role in reducing the incidence of CVD.^6^ And telecardiology in the form of teleconsultation in areas without access to a specialist can potentially optimize identifying patients at higher risk for CVD.

Accorsi et al.,^7^ in the article published in the *Arquivos Brasileiros de Cardiologia*, analyzed data from cardiology teleconsultations performed on patients living in remote cities in the North region of Brazil. According to the last Census of the Brazilian Institute of Geography and Statistics, the North Region of Brazil has more than 12,500,000 inhabitants, with at least 20% living in remote areas, far from urban centers or far from inhabited places, and with difficult access.^4^ The North Region has the country’s lowest density of medical services, with an average of one doctor per thousand inhabitants, but reaching 0.2 per thousand inhabitants in remote areas.^8^

The study^7^ involved the telemedicine center at Hospital Israelita Albert Einstein, having been a reference for 104 face-to-face care centers in the North of Brazil related to the specialized medical assistance program of the Support Program for Institutional Development of the Unified Health System (PROADI) of the Ministry of Health. The patients were previously evaluated by general practitioners in the community who requested a specialist consultation in cardiology. All remote assessments in this program included the patient, the general practitioner at the health facility, and the cardiologist at the telemedicine center in real-time (synchronous). Among the parameters analyzed by the authors are the reason for referral, clinical history and physical examination, and post-telemedicine evaluation regarding tests, medications, diagnoses, and prescriptions. From 02/17/2020 to 10/04/2021, 653 patients were scheduled, with an attendance rate of 85.7%.

Points to be highlighted in the cardiology teleconsultation:

- Main symptoms that motivated the referral: chest pain, dyspnea, palpitation, and syncope.
- In only 26.1%, there was suspicion of ischemic heart disease
- Main risk factors identified: hypertension, dyslipidemia, smoking, sedentary lifestyle, and diabetes mellitus.
- Most patients had a normal physical examination and electrocardiogram.
- All drug prescriptions have been changed to some extent.
- Little need to order additional tests, with very few patients indicated for intervention.
- Many patients needed companions to understand the basic explanations of the treatment.

This was the first study to analyze the characteristics and management of on-demand telemedicine cardiology consultations for low-income populations in remote areas of Brazil, demonstrating the potential of cardiology teleconsultation to optimize referrals to specialists in remote and difficult-to-access areas in North Region and the opportunity to optimize the medical treatment of several heart diseases.
